# Supplementary material for: Outcomes and factors associated with medical treatment failure in patients with spinal epidural abscess: A 14-year experience
Source: PLoS One. 2026 May 5;21(5):e0348373. doi: 10.1371/journal.pone.0348373 (PMC13143116; doi:10.1371/journal.pone.0348373)
Supplement: Attachment — Submitted filename: response to reviewers.docx [file pone.0348373.s003.docx]

**Response to reviewers**:

**Reviewer #1**:

1. Conservative management still appears throughout the manuscript instead of medical management. As noted previously medical management is a better term.

***Response: We have revised the manuscript and changed the term conservative to medical as suggested***.

2. "Patients with medical treatment failure, compared with those without failure and those
who initially underwent surgery, had higher SEA-related mortality (16.0% vs 5.9% vs
0%, p=0.038)." This statement contradicts with "In comparison, 3 patients in the initial intervention group presented criteria for failure (17.6%, p=0.053); 1 due to SEA-related death and 2 due to neurological worsening" when it comes to SEA related mortality.

***Response: Thank you for noticing the error. It was a mistake in writing the sentence. The correct percentages of SEA-related mortality were: 16.0% (4/25) of those with failure to medical treatment, vs 0% (0/34) of those without failure and 5.9% (1/17) of those with initial surgery, as also shown in table 3. The way the sentence was written, it should state: “"Patients with medical treatment failure, compared with those without failure and those who initially underwent surgery, had higher SEA-related mortality (16.0% vs 0% vs 5.9%, p=0.038).". We have changed the sentence.***

3. "There were no other differences in clinical presentation, radiology, microbiology or antibiotic management between the groups." As per the table, other significant differences between the not mentioned in the text include previous stroke, obesity, level of spine involvement and presence of bacteremia. Kindly review this.

***Response: You are correct. We have modified the sentence.***

4. There are a lot of spelling and grammatical errors littered through the document. The English is not standard. This needs to be addressed.

***Response. Thank you for the suggestion. We have now corrected the spelling and grammar of the manuscript according to recommendations of a native English speaker***

**Reviewer #2**:

The authors have adequately addressed all comments raised in the previous review round. In particular, the clarification regarding the management of patients with neurological deficits significantly improves the transparency and clinical interpretation of the study. The inclusion of relevant literature and current consensus recommendations has strengthened the discussion. The manuscript is technically sound, the data are clearly presented, and the conclusions are supported by the results. I have no further comments and support publication.

***Response: Thank you for your revision and the previous comments to the paper. We agree that the response to the comments have improved the quality of the manuscript.***
